# The Features of the Synovium in Early Rheumatoid Arthritis According to the 2010 ACR/EULAR Classification Criteria

**DOI:** 10.1371/journal.pone.0036668

**Published:** 2012-05-04

**Authors:** Marleen G. H. van de Sande, Maria J. H. de Hair, Yvonne Schuller, Gijs P. M. van de Sande, Carla A. Wijbrandts, Huib J. Dinant, Danielle M. Gerlag, Paul P. Tak

**Affiliations:** Division of Clinical Immunology and Rheumatology, Academic Medical Center, University of Amsterdam, Amsterdam, The Netherlands; University of California, San Francisco, United States of America

## Abstract

**Objectives:**

It has been shown in early arthritis cohorts that the 2010 ACR/EULAR criteria for rheumatoid arthritis (RA) enable an earlier diagnosis, perhaps at the cost of a somewhat more heterogeneous patient population. We describe the features of synovial inflammation in RA patients classified according to these new criteria.

**Methods:**

At baseline, synovial tissue biopsy samples were obtained from disease-modifying antirheumatic drug (DMARD)-naïve early RA patients (clinical signs and symptoms <1 year). Synovial tissue was analyzed for cell infiltration, vascularity, and expression of adhesion molecules. Stained sections were evaluated by digital image analysis. Patients were classified according to the two different sets of classification criteria, autoantibody status, and outcome.

**Findings:**

Synovial tissue of 69 RA patients according to 2010 ACR/EULAR criteria was analyzed: 56 patients who fulfilled the criteria for RA at baseline and 13 who were initially diagnosed as undifferentiated arthritis but fulfilled criteria for RA upon follow up. The synovium at baseline was infiltrated by plasma cells, macrophages, and T cells as well as other cells, and findings were comparable to those when patients were selected based on the 1987 ACR criteria for RA. There was no clear cut difference in the characteristics of the synovium between RA patients initially diagnosed as undifferentiated arthritis and those who already fulfilled classification criteria at baseline.

**Conclusion:**

The features of synovial inflammation are similar when the 2010 ACR/EULAR classification criteria are used compared to the 1987 ACR criteria.

## Introduction

Early and aggressive treatment with disease-modifying antirheumatic drugs (DMARDs) is the cornerstone of initial therapy for rheumatoid arthritis (RA). This therapeutic strategy has been shown to halt or prevent disease progression and joint destruction, and thereby improve outcome in RA patients. [1]–[3] To be able to start appropriate treatment for the individual patient, a timely diagnosis and estimation of the prognosis is required.

In the past years efforts have been made to identify clinical and molecular parameters that could aid in the diagnostic and/or prognostic process. [4]–[7] Recently, ACR and EULAR have developed a set of new classification criteria for RA that is used to diagnose early RA. [8], [9] The 2010 ACR/EULAR criteria allow earlier diagnosis of RA, but the clinical picture is slightly different on the group level, and some patients with self-limiting disease may be falsely diagnosed with RA. [8], [10]–[12].

As it can be anticipated that the new criteria will be used for research purposes and since the synovium is the primary target in RA, we wanted to describe the features of synovial inflammation in RA patients classified according to the new 2010 ACR/EULAR criteria for RA compared to the use of the 1987 ACR criteria. Therefore, in a prospective cohort study, we analyzed synovial tissue samples from DMARD-naïve, early RA patients in relationship to the use of the different sets of classification criteria, autoantibody status, and disease outcome after follow up.

## Methods

### Objectives

To analyze synovial tissue samples from DMARD-naïve, early RA patients in relationship to the use of the 1987 ACR RA versus 2010 ACR/EULAR classification criteria, autoantibody status, and disease outcome after follow up.

### Patients

Since 2002, a prospective cohort of early arthritis patients has been gathered at the Academic Medical Center/University of Amsterdam (AMC) in Amsterdam, the Netherlands. This venture aimed at the identification of novel diagnostic and prognostic biomarkers has been termed the ‘Synoviomics project’. [13] The immediate goal of the ‘Synoviomics project’ is to provide insight into the pathogenesis of various forms of arthritis, especially RA. From this cohort we selected all patients who fulfilled the 2010 ACR/EULAR criteria for RA already at baseline or after 2 years follow up [8], [9] and from whom synovial tissue samples were available for analysis. The patients had less than 1 year disease duration, as measured from the first clinical evidence of joint swelling, irrespective of which joint was initially affected. Upon inclusion all patients had active arthritis of at least a wrist, ankle or knee joint. After inclusion patients were treated by their rheumatologist. In case of a clinical diagnosis of RA, DMARD treatment was initiated directly after baseline study procedures were completed. DAS28 was systematically determined and patients were treated according to the treat-to-target principle, aiming for DAS28<2.6. If a combination of DMARDs did not result in a DAS28<3.2 then a biological was started. Upon decision of the treating physician corticosteroids were started in combination with a DMARD, either high dose and tapered down in 6–8 weeks or continuously low dose, to achieve disease remission. The patients with undifferentiated arthritis (UA) were treated with intra-articular steroids, and if arthritis was persistent, a DMARD was started. In the patients with UA at baseline and after follow-up (according to the 1987 criteria) 7 patients were started on DMARD treatment and this was continued during follow-up in 6 patients.

### Ethics

The study was approved by the Medical Ethics Committee of the Academic Medical Center/University of Amsterdam and performed according to the Declaration of Helsinki. All patients gave written informed consent.

### Study Design

At baseline, arthroscopic synovial biopsy samples [14] as well as demographic and clinical assessment data were obtained. At baseline and after 2 years of follow up a diagnosis was made according to the 2010 ACR/EULAR criteria (8;9) and according to the 1987 ACR criteria for RA. [15] After follow up, patients were also classified according to disease outcome as having self-limiting disease, persistent non-erosive disease, or persistent erosive disease. [4] Self-limiting arthritis was defined as absence of arthritis on examination after follow up, in a patient who had not taken DMARDs or steroids in the preceding 3 months. Presence of arthritis in at least one joint and/or treatment with DMARDs or steroids within the previous 3 months was defined as persistent disease. Joint destruction was evaluated by the presence or absence of erosions and joint space narrowing on X-rays of hands and feet (defined by a score of ≥1 on the Sharp-van der Heijde erosion/joint space narrowing score scale [16]).

### Disease Activity Parameters

Presence of IgM rheumatoid factor (IgM-RF) and anti-citrullinated protein antibodies (ACPA) was measured by IgM-RF ELISA (Sanquin, Amsterdam, the Netherlands) and anti-CCP2 ELISA (CCPlus, Eurodiagnostica, Nijmegen, the Netherlands), respectively.

Patient’s visual analog scale (VAS) for global disease activity (scale 0–100 mm), VAS for pain (scale 0–100 mm), 68 tender joint count (TJC68) and 66 swollen joint count (SJC66), morning stiffness in minutes, erythrocyte sedimentation rate (ESR), and serum levels of C-reactive protein (CRP) were used to evaluate disease activity.

### Synovial Biopsy Collection, Immunohistochemical Staining and Quantification

All individuals underwent arthroscopic synovial tissue sampling of an inflamed wrist, knee or ankle joint. At least six specimens were collected for immunohistochemistry, as previously described [17], to correct for sampling error. The synovial biopsy samples were snap-frozen en bloc in Tissue-Tek OCT (Miles, Elkhart, IN) immediately after collection. Sections (5 µm each) were cut and mounted on Star Frost adhesive glass slides (Knittelgläser, Braunschweig, Germany). Sealed slides were stored at −80°C until further use.

Synovial tissue sections were stained in one session specifically for this study using the following monoclonal antibodies: anti-CD3 (SK7; Becton Dickinson, San Jose, CA) to detect T cells, anti-CD22 (CLB-B-ly/1, 6B11; Central Laboratory of the Netherlands Red Cross Blood Transfusion Service, Amsterdam, the Netherlands) for B cells, anti-CD55 (67; Serotec, Oxford, United Kingdom) for fibroblast-like synoviocytes (FLS), anti-CD68 (EBM11; Dako, Glostrup, Denmark) for macrophages, anti-CD138 (B-B4; Immunotech, Marseille, France) for plasma cells, anti-tryptase for mast cells (AA1; Dako), anti-von Willebrand factor (vWF; F8/86; Dako) for blood vessels, anti-CD106/vascular cell adhesion molecule-1 (VCAM-1; 1G11B1; Sanbio, Uden, the Netherlands), and anti-vascular endothelial growth factor (VEGF; C1; Santa Cruz Biotechnology, Santa Cruz, CA).

A three-step immunoperoxidase protocol was used to detect specific staining for all phenotypic markers and vWF. [18] For VCAM-1 and VEGF, biotinylated tyramine was used for amplification, as previously described. [18], [19] As a negative control, irrelevant/isotype-matched immunoglobulins were applied to the sections instead of the primary antibody or the primary antibody was omitted. Expression of the synovial biomarkers was quantified by digital image analysis within one week after staining, as previously described. [20], [21] For each marker 18 representative high power fields (2.2 mm^2^) were analyzed. Digital image analysis was performed by 3 trained observers (MS, YS, GS) blinded for clinical classification. Expression levels of CD3, CD22, CD55, CD68, CD138, and tryptase are presented as count/mm^2^; vWF, VEGF, and VCAM-1 expression levels are presented as integrated optical density (IOD)/mm^2^, an arbitrary unit representing the intensity of staining per mm^2^. [22].

### Statistical Analysis

Continuous data are described as median and interquartile range (IQR). To compare baseline patient characteristics and expression of biomarkers between the different RA subgroups, the Kruskal-Wallis test was used when more than 2 groups were compared; subsequently the Mann-Whitney U test was used to compare differences between two subgroups. Nominal data were represented as percentages and analyzed using the Chi^2^-test. All statistical analyses were performed using SPSS v16.0 software (SPSS, Chicago, IL). A *P*-value of <0.05 was considered statistically significant.

## Results

### Patients

Synovial tissue samples were available from 69 early RA patients according to the 2010 ACR/EULAR classification criteria. Fifty-six of these patients fulfilled the criteria at baseline and after follow up (RA-RA). Twelve of these patients were not available for follow up and were therefore excluded from the outcome analysis. Thirteen patients were initially classified as UA, but fulfilled the 2010 ACR/EULAR ACR criteria after 2 years of follow up (UA-RA). This resulted in 69 RA patients for whom expression of synovial biomarkers at baseline could be related to diagnosis and autoantibody status, and 57 patients for whom synovial biomarkers could also be related to clinical outcome after 2 years.

**Figure 1:**
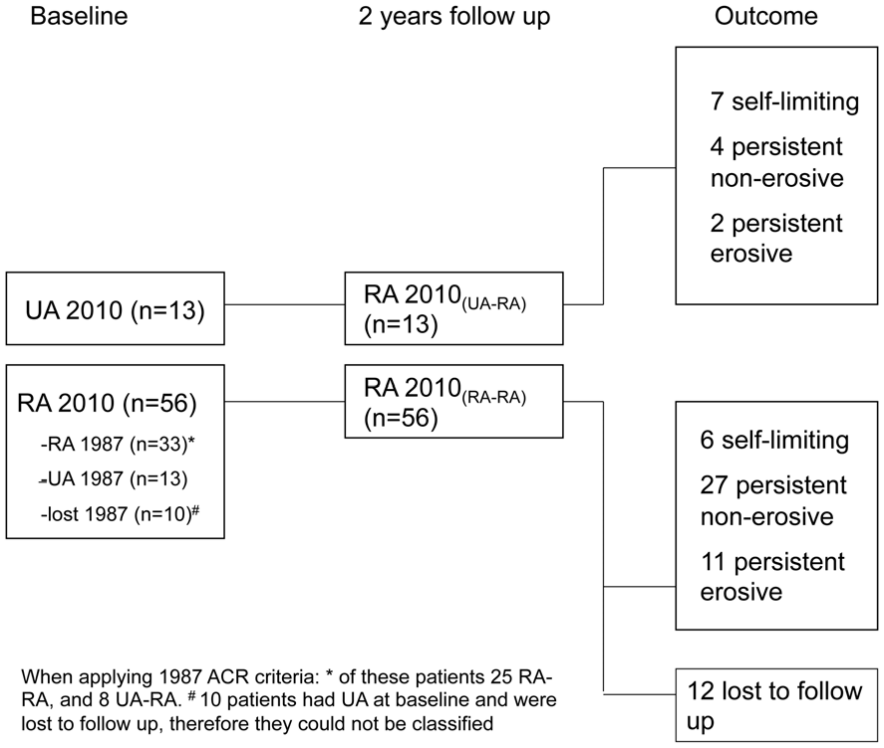
Patient classification. Patient classification at baseline and after 2 years follow up according to 2010 ACR/EULAR criteria, and according to outcome after 2 years follow up.

Disease activity parameters and age were significantly different between the RA-RA and UA-RA groups (Table 1). The overall median (IQR) disease duration was 4 (6) months. Thirty-three patients who fulfilled the 2010 ACR/EULAR criteria at baseline also fulfilled the 1987 ACR criteria for RA after follow-up. In the RA-RA group with completed follow up data (n = 44) 6 patients had self-limiting disease, 27 had persistent non-erosive disease, and 11 had persistent erosive disease. In the UA-RA group (n = 13) 7 patients had self-limiting disease, 4 had persistent non-erosive disease and 2 had persistent erosive disease (see Figure 1).

**Table 1:** Baseline patient characteristics.

	RA ACR/EULAR 2010			*P*-value
	RA (all) (n = 69)	RA-RA (n = 56)	UA-RA (n = 13)	
**Age (yrs)**	48 (169)	47 (18)	57 (18)	**0.03**
**Dis.dur. (mo)**	4 (6)	5 (7)	4 (4)	0.16
**Female, n (%)**	49 (71%)	40 (71%)	9 (69%)	0.89
**VAS global disease activity (0–100 mm)**	50 (42)	56 (39)	43 (49)	0.14
**VAS pain (0–100 mm)**	51 (50)	63 (46)	30 (38)	**0.04**
**MS (min)**	30 (55)	45 (75)	1 (15)	0.17
**ESR (mm/h)**	27 (30)	29 (31)	14 (32)	0.09
**CRP (mg/L)**	11 (24)	12 (30)	5 (16)	**0.02**
**TJC68 (n)**	9 (16)	12 (18)	1 (2)	**<0.001**
**SJC66 (n)**	5 (8)	7 (7)	1 (1)	**<0.001**
**RF pos, n (%)**	24 (35%)	21 (38%)	3 (20%)	**0.05**
**ACPA pos, n (%)**	23 (30%)	23 (41%)	0 (0%)	**0.008**

Data are presented as median (interquartile range) or number (n [%]), as appropriate. Baseline characteristics were compared between the two diagnostic groups using a Mann-Whitney U test or a Chi2-test (sex, RF pos, ACPA pos). A *P*-value of <0.05 was considered statistically significant (bold). RA = rheumatoid arthritis; RA (all) = all patients with RA diagnosis after 2 years of follow up; RA−RA = RA at baseline and follow up; UA−RA = initially UA, but definitive diagnosis of RA at follow up; dis.dur. = disease duration; VAS = visual analog scale; MS = morning stiffness; ESR = erythrocyte sedimentation rate; CRP = C-reactive protein; TJC68 = tender joint count; SJC66 = swollen joint count; RF = IgM rheumatoid factor; ACPA = anti-citrullinated protein antibodies; pos = serum positive.

The characteristics of the synovium in RA patients according to the 2010 ACR/EULAR classification criteria are similar to those when the 1987 ACR criteria are used.

**Figure 2:**
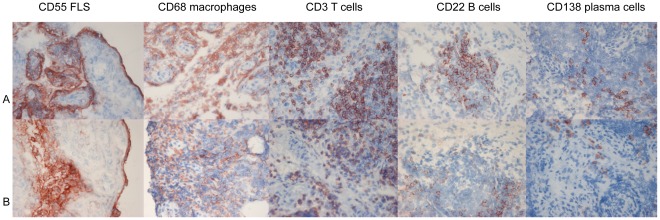
Synovial tissue expression of different cellular markers. Synovial tissue expression of, CD55+ fibroblast-like synoviocytes (FLS), CD3+ T CD68+ macrophages, CD3+ T cells, CD22+ B cells, CD138+ plasma cells. A:RA patient according to the 1987 ACR criteria, B:RA patient according to the 2010 ACR/EULAR criteria.

First, we examined the features of synovial inflammation in the patients who fulfilled the 2010 ACR/EULAR criteria for RA at baseline but not the 1987 criteria for RA (n = 23) in comparison to patients who fulfilled both the 2010 ACR/EULAR criteria and the 1987 criteria for RA after 2 years of follow up (n = 33) (since the 1987 ACR criteria have been developed to classify RA in more advanced disease, we used this as gold standard for RA in this analysis). In the patients fulfilling only the 2010 ACR/EULAR criteria for RA there was interindividual variability, but on average the synovium was characterized by marked infiltration with macrophages, T cells, plasma cells, B cells and mast cells, increased numbers of synovial fibroblasts, hypervascularity, and overexpression of VCAM-1 and VEGF (See Figure 2). The results were similar to those from RA patients according to the 1987 ACR criteria after 2 years follow up (Table 2).

**Table 2:** Expression of synovial phenotypic and vascular markers in patients who fulfilled 2010 ACR/EULAR criteria for RA at baseline after follow up and in the patients who fulfilled1987 ACR criteria.

	RA 2010 (n = 23)	RA 1987 (n = 33)	*P*-value
**CD3 (count/mm^2^)**	201(856)	198(795)	0.86
**CD55 (count/mm^2^)**	1,144 (1,253)	1,163 (1,365)	0.89
**CD68L (count/mm^2^)**	274(275)	234 (396)	0.84
**CD68SL (count/mm^2^)**	571 (1,054)	393 (1562)	0.92
**CD22 (count/mm^2^)**	170 (470)	214 (330)	0.94
**CD138 (count/mm^2^)**	53 (504)	139 (564)	0.98
**Tryptase (count/mm^2^)**	235 (445)	338(345)	0.13
**vWF (IOD/mm^2^)**	205,845 (175,450)	171,078 (167,211)	0.75
**VEGF (IOD/mm^2^)**	59,435 (157,527)	63,461 (81,425)	0.86
**VCAM-1 (IOD/mm^2^)**	119,676 (298,344)	184,267 (265,919)	0.70

Values are presented as median (interquartile range). A Mann-Whitney U test was used to compare the different diagnostic groups; a *P*-value of <0.05 was considered statistically significant. RA = rheumatoid arthritis. CD3 refers to T cells; CD55– fibroblast-like synoviocytes; CD68– macrophages; CD22– B cells; CD138– plasma cells; tryptase – mast cells. L = intimal lining layer; SL = synovial sublining; vWF = von Willebrand factor; VEGF = vascular endothelial growth factor; VCAM-1 = vascular cell adhesion molecule-1. IOD = integrated optical density.

Second, we selected the patients in our cohort who fulfilled the 2010 ACR/EULAR criteria for RA at baseline (n = 56). In these patients we subsequently applied the 1987 ACR criteria for RA and analyzed if there were differences between the patients classified as UA at baseline and after follow up (UA-UA) (n = 13), UA at baseline and as RA after follow up (UA-RA) (n = 8) or RA at baseline and after follow up (RA-RA) (n = 25). Of these 56 patients, 10 patients had UA at baseline according to the 1987 criteria but were lost to follow up and therefore could not be given a definite diagnosis. They were excluded from this analysis. No statistically significant differences were observed in the numbers of CD3 positive T cells (*P* = 0.28), CD55 positive FLS (*P* = 0.62), CD68 positive intimal macrophages (*P* = 0.81), CD68 positive macrophages in the synovial sublining (*P* = 0.91), CD22 positive B cells (*P* = 0.58), CD138 positive plasma cells (*P* = 0.48), tryptase positive mast cells (*P* = 0.61), or expression of vWF (*P* = 0.21), VEGF (*P* = 0.99), and VCAM-1 (*P* = 0.33) between the 3 patients groups (Table 3). Together, these data clearly show that the features of synovial inflammation are on average similar when the 2010 ACR/EULAR classification criteria are used compared to the 1987 ACR criteria.

**Table 3:** Expression of synovial phenotypic and vascular markers in patients who fulfilled 2010 ACR/EULAR criteria for RA at baseline subclassified according to fulfillment of 1987 ACR criteria at baseline and after follow up.

	RA 2010 ACR/EULAR (n = 46)			*P*-value
	RA-RA 1987 (n = 25)	UA-RA 1987 (n = 8)	UA-UA 1987 (n = 13)	
**CD3 (count/mm^2^)**	327 (867)	107 (157)	304 (882)	0.28
**CD55 (count/mm^2^)**	1,163 (1,273)	1,514 (1,914)	963 (1,116)	0.62
**CD68L (count/mm^2^)**	256 (458)	139 (150)	229 (456)	0.81
**CD68SL (count/mm^2^)**	491 (1,611)	362 (476)	570 (1,211)	0.91
**CD22 (count/mm^2^)**	198 (389)	233 (286)	407 (598)	0.58
**CD138 (count/mm^2^)**	191 (585)	30 (329)	76 (409)	0.48
**Tryptase (count/mm^2^)**	337 (311)	419 (549)	221 (587)	0.61
**vWF (IOD/mm^2^)**	205,558 (161,492)	93397(181,405)	224,825 (194,340)	0.21
**VEGF (IOD/mm^2^)**	63,659 (96,163)	57,865 (59,901)	55,365 (140,674)	0.99
**VCAM-1 (IOD/mm^2^)**	195,424 (302,882)	96,545 (175,238)	184,647 (322,252)	0.33

Values are presented as median (interquartile range). A Kruskal-Wallis test was used to compare the different diagnostic groups; a *P*-value of <0.05 was considered statistically significant. RA = rheumatoid arthritis. UA = undifferentiated arthritis. CD3 refers to T cells; CD55– fibroblast-like synoviocytes; CD68– macrophages; CD22– B cells; CD138– plasma cells; tryptase – mast cells. L = intimal lining layer; SL = synovial sublining; vWF = von Willebrand factor; VEGF = vascular endothelial growth factor; VCAM-1 = vascular cell adhesion molecule-1. IOD = integrated optical density.

Phenotypic and vascular synovial tissue markers do not define autoantibody status or outcome in RA patients.

To investigate if the inflammatory changes in the synovium are related to a specific subset of RA patients, we analyzed the expression of different phenotypic and vascular markers in RA patients with and without elevated serum levels of RA specific autoantibodies. Second, we compared the expression of these markers in RA patients who developed self-limiting, persistent or persistent erosive disease after follow up.

No difference in expression of phenotypic or vascular markers was observed when comparing RA patients according to the 2010 ACR/EULAR criteria for RA upon 2 years follow up with elevated IgM-RF and/or ACPA levels compared to RA patients who were autoantibody negative (CD3 positive T cells (*P* = 0.61), CD55 positive FLS (*P* = 0.29), CD68 positive intimal macrophages (*P* = 0.73), CD68 positive macrophages in the synovial sublining (*P* = 0.91), CD22 positive B cells (*P* = 0.63), CD138 positive plasma cells (*P* = 0.69), tryptase positive mast cells (*P* = 0.60), vWF (*P* = 0.31), VEGF (*P* = 0.20) and VCAM-1 (*P* = 0.91). Similarly, no differences were observed when comparing ACPA positive RA with ACPA negative RA patients or IgM-RF positive RA with IgM-RF negative RA patients (data not shown).

Of the 69 patients who fulfilled the 2010 ACR/EULAR criteria for RA after 2 years of follow up, 57 patients could be classified according to outcome. Thirteen patients had self-limiting disease, 31 patients had persistent non-erosive disease and 13 patients had persistent, erosive disease. No statistically significant differences in cell infiltration, vascularity, and expression of adhesion molecules were observed between the different outcome groups (Table 4). Together, these data show that the synovial tissue markers cannot reliably differentiate between the subgroups of RA patients.

**Table 4:** Expression of phenotypic and vascular markers in synovial tissue of RA patients with self-limiting, persistent non-erosive or persistent erosive disease.

	Self-limiting (n = 13)	Persistent non-erosive(n = 31)	Persistent erosive (n = 13)	*P*-value
**CD3 (count/mm^2^)**	210 (327)	209 (561)	131 (907)	0.68
**CD55 (count/mm^2^)**	1,329 (859)	929 (1,474)	1319 (1,218)	0.50
**CD68L (count/mm^2^)**	305 (528)	234 (442)	218 (277)	0.57
**CD68SL (count/mm^2^)**	604 (1,417)	588 (1,137)	268 (712)	0.24
**CD22 (count/mm^2^)**	135 (300)	232 (412)	224 (383)	0.78
**CD138 (count/mm^2^)**	106 (276)	68 (205)	78 (730)	0.53
**Tryptase (count/mm^2^)**	364 (737)	330 (395)	339 (369)	0.76
**vWF (IOD/mm^2^)**	250,901 (150,431)	174,116 (136,053)	119,011 (202,828)	0.10
**VEGF (IOD/mm^2^)**	50,685 (53,362)	82,024 (180,150)	79,926 (73,768)	0.30
**VCAM-1 (IOD/mm^2^)**	95,055 (302,693)	187,968 (302,271)	150,069 (275,806)	0.98

Values are presented as median (interquartile range). A Kruskal-Wallis test was used to compare the different diagnostic groups; a *P*-value of <0.05 was considered statistically significant (bold). CD3 refers to T cells; CD55– fibroblast-like synoviocytes; CD68– macrophages; CD22– B cells; CD138– plasma cells; tryptase – mast cells. L = lining; SL = sublining; vWF = von Willebrand factor; VEGF = vascular endothelial growth factor; VCAM-1 = vascular cell adhesion molecule-1. IOD = integrated optical density.

## Discussion

With the emergence of the 2010 ACR/EULAR criteria more patients are classified as having RA than with the 1987 ACR RA criteria in early arthritis cohorts. [10]–[12] This enables the diagnosis of RA in patients presenting in early arthritis clinics with a potentially destructive form of inflammatory arthritis. However, with these novel 2010 ACR/EULAR criteria not only patients with a persistent and destructive form of RA requiring aggressive DMARD therapy are identified but also some patients with a self-limiting disease. [10] There has been a recent upsurge in scientific studies of the primary target of RA, the synovium. As one might wonder if RA according to the 2010 ACR/EULAR criteria on average refers to the same disease process with the same features of synovial inflammation as RA according to the 1987 ACR criteria, we sought to describe these in early RA patients according to the new classification criteria.

We observed that the synovium of RA patients classified according to the 2010 ACR/EULAR criteria at baseline is infiltrated by macrophages, plasma cells, and T cells as well as other cells like B cells, mast cells, and FLS. In addition, there was overexpresison of VEGF and VCAM-1, and increased vascularity. Findings were on average comparable if RA patients were selected based on the 1987 ACR criteria for RA. When we subdivided the patients who fulfilled the 2010 criteria into patients who would have been classified as UA according to the 1987 ACR criteria or only would have fulfilled the 1987 ACR criteria after follow up, and those who fulfilled the 1987 ACR criteria already at baseline, the synovial tissue infiltrate was similar in the three subsets of patients. This shows that when applying these new criteria on average similar synovial inflammatory changes are observed compared to the 1987 ACR criteria for RA. Only few patients (n = 2 [5%]) who were classified as RA according to the 1987 criteria did not fulfill the 2010 criteria at baseline, which is consistent with previous studies. [10], [11].

The results presented here also independently confirm previous work, showing that the features of synovial inflammation are similar between autoantibody positive and autoantibody negative RA [23]–[25] although such differences have initially been suggested. [26] The difference in the latter study might perhaps be explained by differences in disease activity between the two groups, as patients with ACPA positive RA had on average higher levels of disease activity in that study.

Our findings do not support the notion that synovial tissue infiltrate analysis will play an important role in guidance of treatment decisions in individual early RA patients, as there was no clear cut difference in baseline features of the synovium between patients with self-limiting, persistent non-erosive and persistent erosive disease. We cannot exclude the possibility that on the group level statistically significant differences might be found in larger studies or if different disease controls would be included, but to be useful in clinical practice high predictive values for a specific disease outcome would be needed. In fact, with a different selection of disease controls, we did observe statistically significant differences between diagnostic groups (early RA versus a mixed early non-RA group) on the group level in a previous study. [7] However, at this moment none of the available tests would justify the routine use of synovial biopsy in clinical practice to establish the diagnosis or outcome of RA, except for specific cases of for instance infection, crystal induced arthritis and neoplasms. [27].

In conclusion, the characteristics of the synovium are on average similar in patients classified according to the 2010 ACR/EULAR criteria for RA compared to those when the 1987 ACR criteria are used. This information is important for the interpretation of future scientific studies of the synovium using the new classification criteria.
